# Spinal subdural hematoma and subdural anesthesia following combined spinal–epidural anesthesia: a case report

**DOI:** 10.1186/s12871-021-01352-3

**Published:** 2021-04-26

**Authors:** Yanmei Bi, Junying Zhou

**Affiliations:** 1 Department of Anesthesiology, West China Second University Hospital of Sichuan University, Sichuan Province Chengdu, China; 2 Key Laboratory of Birth Defects and Related Diseases of Women and Children (Sichuan University), Ministry of Education, Sichuan Province Chengdu, China; 3 Department of Operation Room, West China Second University Hospital of Sichuan University, Sichuan Province Chengdu, China

**Keywords:** Spinal subdural hematoma, Subdural anesthesia, Combined spinal‐epidural anesthesia

## Abstract

**Background:**

Subdural anesthesia and spinal subdural hematoma are rare complications of combined spinal-epidural anesthesia. We present a patient who developed both after multiple attempts to achieve combined spinal–epidural anesthesia.

**Case presentation:**

A 21-year-old parturient, gravida 1, para 1, with twin pregnancy at gestational age 34^+ 5^ weeks underwent cesarean delivery. Routine combined spinal–epidural anesthesia was planned; however, no cerebrospinal fluid outflow was achieved after several attempts. Bupivacaine (2.5 mL) administered via a spinal needle only achieved asymmetric blockade of the lower extremities, reaching T12. Then, epidural administration of low-dose 2-chlorprocaine caused unexpected blockade above T2 as well as tinnitus, dyspnea, and inability to speak. The patient was intubated, and the twins were delivered. Ten minutes after the operation, the patient was awake with normal tidal volume. The endotracheal tube was removed, and she was transferred to the intensive care unit for further observation. Postoperative magnetic resonance imaging suggested a spinal subdural hematoma extending from T12 to the cauda equina. Sensory and motor function completely recovered 5 h after surgery. She denied headache, low back pain, or other neurologic deficit. The patient was discharged 6 days after surgery. One month later, repeat MRI was normal.

**Conclusions:**

All anesthesiologists should be aware of the possibility of SSDH and subdural block when performing neuraxial anesthesia, especially in patients in whom puncture is difficult. Less traumatic methods of achieving anesthesia, such as epidural anesthesia, single-shot spinal anesthesia, or general anesthesia should be considered in these patients. Furthermore, vital signs and neurologic function should be closely monitored during and after surgery.

## Background

Combined spinal–epidural anesthesia (CSE) is a commonly performed anesthesia method for cesarean delivery that is safe and effective. However, subdural anesthesia and spinal subdural hematoma (SSDH) are rare potential complications. SSDH may be caused by many factors, including difficulties during dural puncture, coagulation disorders, and increased intra-abdominal pressure [1–4]. Subdural anesthesia is also related to difficulties with dural puncture [5]. We report a patient who developed SSDH and subdural anesthesia after several attempts to achieve CSE.

## Case presentation

A 21-year-old parturient (weight, 55 kg; height, 155 cm), gravida 1, with twin pregnancy at 34^+ 5^ weeks was scheduled to undergo planned cesarean delivery because of intrahepatic cholestasis of pregnancy (ICP). Her past medical history was unremarkable. She was not taking anticoagulants. Laboratory testing results were as follows: hemoglobin (Hb), 135 g/L; platelet count, 147 × 10^9^/L; prothrombin time (PT), 12.5 s; activated partial thromboplastin time (APTT), 26.9 s; international normalized ratio (INR), 1.18; total bile acid (TBA), 21.3 umol/L; alanine aminotransferase (ALT) 78 U/L; aspartate aminotransferase (AST) 84 U/L. Base line heart rate was 79 beats/min, respiratory rate was 18 breaths/min, and blood pressure (BP) was 120/67 mm Hg.

**Fig. 1:**
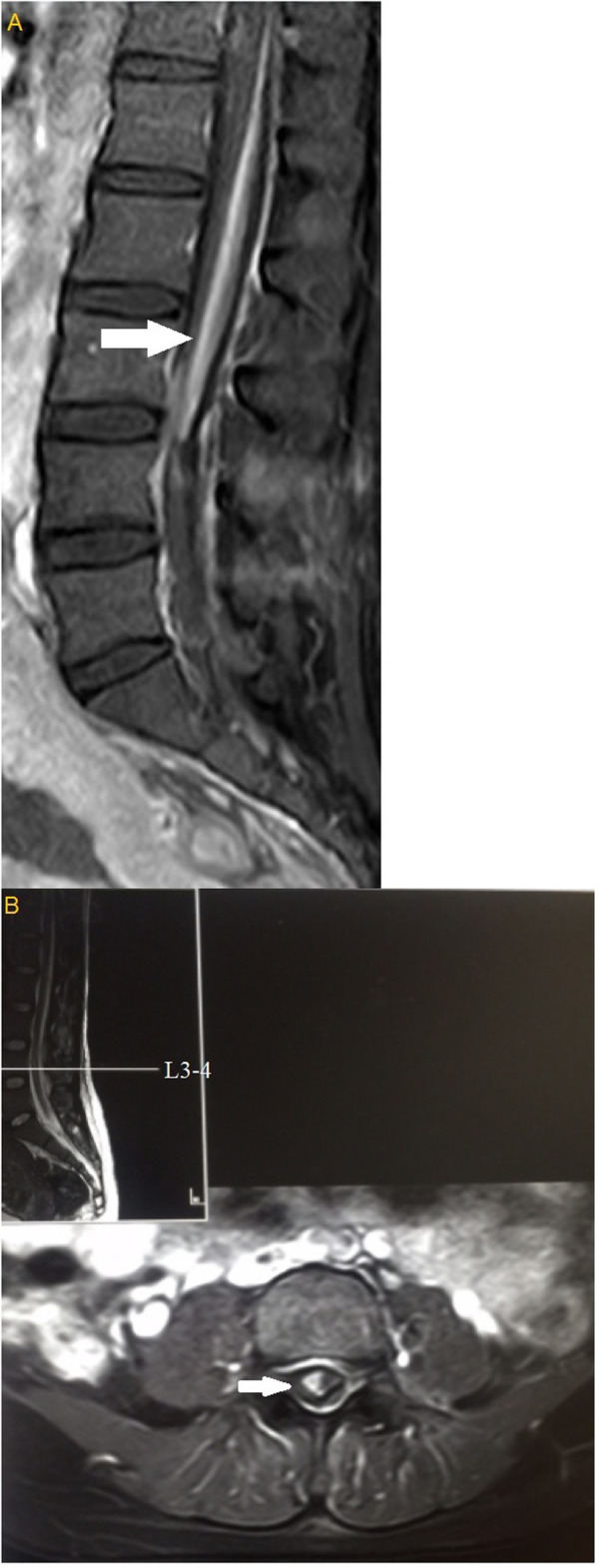
Magnetic resonance imaging of the lumbar spine after cesarean delivery (**a**, sagittal section; **b**, axial). White arrows indicate the spinal subdural hematoma

CSE was attempted at the L3–4 intervertebral space using an 18G Tuohy needle (ZHEJIANG HAISHENG MEDICAL DEVICE CO., LTD). After loss of resistance to air, access to the epidural space was presumed and a 25G Sprotte needle was inserted; however, cerebrospinal fluid (CSF) outflow was not obtained. A second senior anesthesiologist then attempted to repeat puncture at the L3–4 level but failed to obtain CSF outflow. An attempt was made at the L2–3 interspace: breakthrough was felt with the spinal needle but CSF outflow was still not obtained. We considered that a “valve” may have formed because of the needle design, causing poor CSF outflow. Furthermore, puncture was difficult. Therefore, a trial dose of bupivacaine (2.5 mL, 0.5 % plain) was administered via the spinal needle to verify subarachnoid space access. This produced only asymmetric blockade. The left side was blocked up to T10 with almost no blockade on the right. Next, a trial dose of epidural lidocaine (3 mL, 1.5 % with 1/200,000 epinephrine) was administered, which resulted in no evidence of local anesthetic toxicity or total spinal anesthesia. Epidural 2-chlorprocaine (5 mL, 3 %) was then administered, which caused an unexpectedly high blockade above T2 as well as tinnitus, difficulty breathing, and inability to speak. The patient’s SpO_2_ gradually decreased and mask-assisted ventilation was initiated. However, the SpO_2_ continued decreasing to 68 % even with 100 % oxygen. Propofol 100 mg and succinylcholine 100 mg were administered intravenously and a 6.5 mm internal diameter endotracheal tube was inserted under video laryngoscopic guidance. Her SpO_2_ increased to 100 % and the twins were delivered. Intraoperative blood loss was 400 mL and 1000 mL of crystalloid fluid was infused. However, the patient’s BP decreased to 80/40 mm Hg a few minutes before surgery was completed, which responded to repeated administration of phenylephrine. Ten minutes after the operation, the patient was awake with a normal tidal volume and the endotracheal tube was removed. Her level of anesthesia remained above T2 and she was unable to move her hands. SpO_2_ remained at 97 % with face mask oxygen and she was transferred to the intensive care unit for further observation. Two hours after surgery, sensation and movement in her right leg had recovered; however, her left leg remained numb and paralyzed. Left leg sensation and motor function completely recovered 3 h later. She denied headache, low back pain, or other neurologic deficit. It was unclear why sensorimotor recovery was significantly slower on the left. Magnetic resonance imaging (MRI) suggested a small (no more than 3 mL) subdural hematoma extending from T12 to the cauda equina (Fig. 1). Postoperative PT, APTT, and INR were 23.4 s, 27 s, and 1.21, respectively. Sensory examination was normal. A neurosurgical consultant recommended in-hospital observation as hematoma absorption was expected. The patient was discharged 6 days after surgery. One month later, repeat MRI was normal.

## Discussion and conclusions

Subdural anesthesia is a well-known but often poorly recognized complication of neuraxial anesthesia/analgesia. The reported incidence of subdural block when attempting to administer neuraxial anesthesia ranges from 0.024 to 0.82 % [6, 7]. Currently, there is no consensus regarding diagnostic criteria for subdural block. According to Lubenow et al. [7], the two major criteria are negative suction test and unexpected widespread sensory blockade; the three minor criteria are > 10 min delay in onset, variable motor block despite small doses of local anesthetic, and extensive sympatholysis with small doses of local anesthetic. The diagnosis can be made when both major criteria and at least one minor criterion are present. Evidence suggesting our patient developed subdural anesthesia included lack of CSF outflow; bilateral asymmetrical sensory and motor block of the lower extremities after administration of 2.5 mL 0.5 % bupivacaine, blockade level above T2 after administration of 3 mL 2 % lidocaine and 5 mL 2-chlorprocaine through an epidural catheter, and development of respiratory depression and moderate hypotension.

Postoperative MRI in our patient showed a small SSDH, an extremely rare and potentially life-threatening complication of spinal and epidural anesthesia. Patients with SSDH usually present with acute back or radicular pain. Prognosis depends on SSDH location and symptom duration. SSDH and subdural anesthesia may have developed because of several possible reasons. First, spontaneous SSDH may have occurred prior to the lumbar puncture. The presence of such a hematoma would have caused the subdural compartment to expand, making it difficult for a spinal needle to reach the subarachnoid space. The patient had ICP, which is the most common liver-related complication of pregnancy and characterized by abnormal liver function and fat malabsorption; the associated fat malabsorption may cause vitamin K deficiency [8]. Vitamin K is a cofactor responsible for synthesis of coagulation factors II, VII, IX, and X. Therefore, prolonged PT and postpartum hemorrhage might be expected in a patient with ICP. Yarnell et al. previously reported an epidural hematoma after epidural catheterization in a pregnant patient with ICP [9]. Thus, a spontaneous subdural hematoma may have occurred prior to lumbar puncture. Second, SSDH may be caused by repeated CSE attempts, as it has been previously reported in patients following difficulty in performing dural puncture [1]. Epidural or spinal (subarachnoid) needle insertion can injure the bridging blood vessels between the dura mater and arachnoid mater. In our patient, postoperative MRI clearly showed that the SSDH was located near the sites of attempted puncture at L3–4 and L2–3. Therefore, we believe that the SSDH was more likely caused by CSE. The hematoma would have caused the subdural compartment to expand, making it difficult for a spinal needle to reach the subarachnoid space [10]. Thus, the bupivacaine was probably injected directly into the subdural space, causing subdural anesthesia. Third, the possibility of subdural catheterization cannot be ruled out. Insufficient local anesthetic was administered to our patient via the epidural route to produce a subdural block. Spinal subdural hemorrhage may have resulted from subdural catheterization.

In retrospect, several things should have been done differently after several unsuccessful CSE attempts at different intervertebral levels. First, the most important thing is that administered bupivacaine to assumed subarachnoid space via spinal needle is inappropriate in the absence of CSF outflow. Which may cause some potential risks, such as incomplete block or failure, subdural anesthesia. So, local anesthetic should not be administered via spinal needle if there is no CSF outflow. Then, CSE attempts should have ceased and general anesthesia, epidural anesthesia, or single-shot spinal anesthesia (SSS) been considered. Generally, patients with a difficult puncture or failure of regional anesthesia are converted to general anesthesia. However, the risk of difficult intubation is increased in the parturient compared with that in the general surgical population. Furthermore, maternal mortality of general anesthesia is 1.7-fold greater than regional anesthesia [11] and general anesthesia prolongs postoperative hospital stay [12]. In our patient, we believed that the Tuohy needle had reached the epidural space, and therefore epidural anesthesia may have been preferable. This would have avoided puncture injury caused by repeated CSE attempts.

**Fig. 2:**
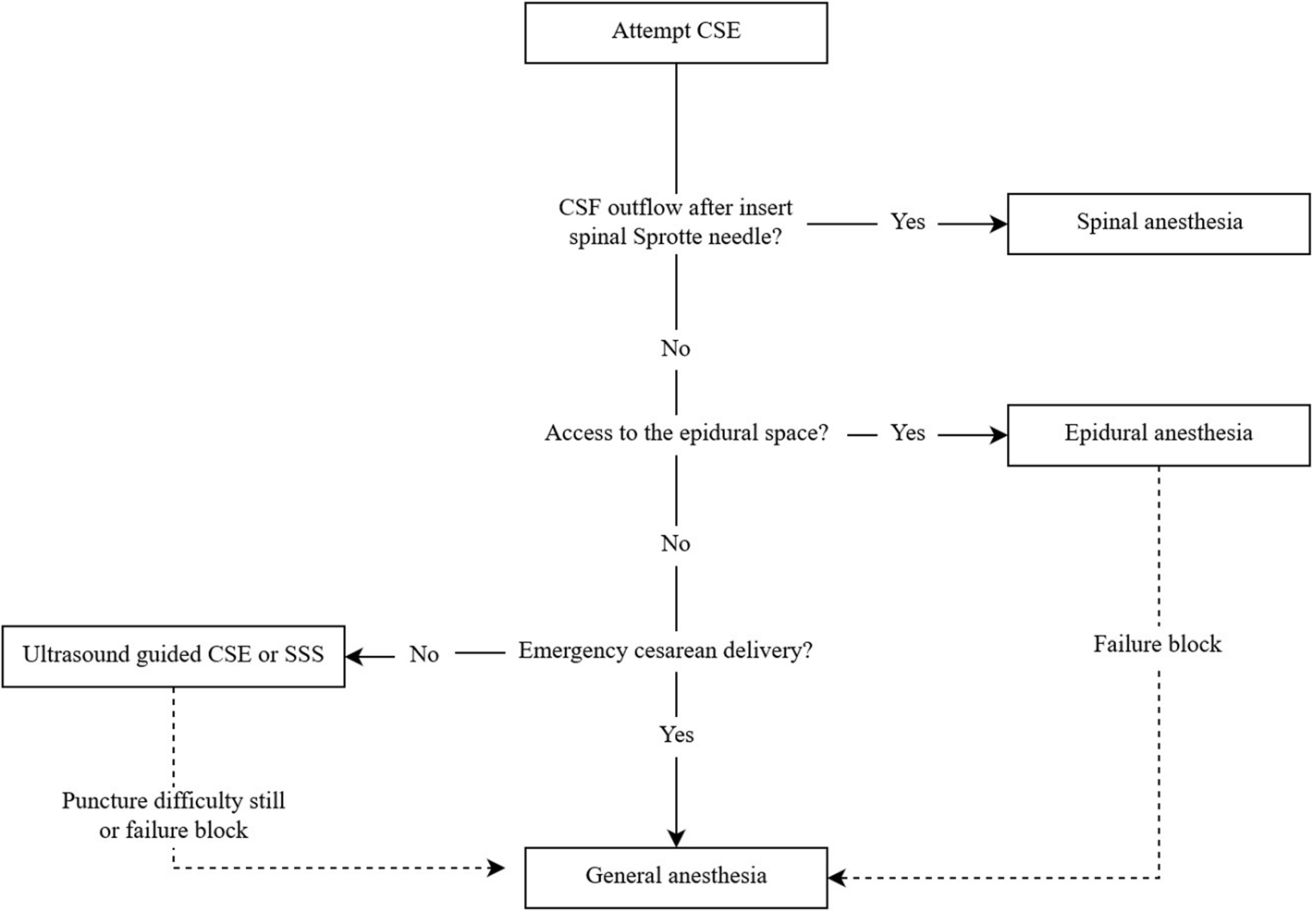
Flow chart after CSE failure

SSS could also have been considered. With SSS, fewer patients experience paresthesias upon spinal needle insertion [13], there is no risk of accidental dural puncture causing post-puncture headache [14], and the incidence of neuraxial hematoma is lower compared with that with CSE [15]. However, more attempts are needed to establish spinal anesthesia with SSS than that with CSE [16]. Lumbar ultrasonography can be used to guide spinal puncture, which reduces the number of puncture attempts and shortens procedure duration [17]. However, ultrasound-guided spinal puncture can still be difficult in cesarean patients and conversion to general anesthesia may be required (Fig. 2).

Patients whose punctures have been difficult should be carefully monitored after surgery for changes in vital signs, anesthesia level subsidence, and development of new symptoms such as back pain or headache. If an intraspinal lesion is suspected, MRI should be performed promptly for diagnosis. SSDH appears as a space-occupying lesion contained within the dura mater and is usually located ventrally [18].

All anesthesiologists should be aware of the possibility of SSDH and subdural block when performing neuraxial anesthesia, especially in patients in whom puncture is difficult. Less traumatic methods of achieving anesthesia, such as epidural anesthesia, SSS, or general anesthesia should be considered in these patients. Furthermore, vital signs and neurologic function should be closely monitored during and after surgery.

## Data Availability

All data related to this case report are contained within the manuscript.

## Abbreviations

CSE: Combined spinal-epidural anesthesia
SSDH: Spinal subdural hematoma
CSF: Cerebrospinal fluid
Hb: Hemoglobin
PT: Prothrombin time
APTT: Activated partial thromboplastin time
INR: International normalized ratio
TBA: Total bile acid
ALT: Alanine aminotransferase
AST: Aspartate aminotransferase
MRI: Magnetic resonance imaging
BP: Blood pressure
SSS: Single-shot spinal anesthesia
